# Supplementary material for: The risk of ergonomic injury across surgical specialties
Source: PLoS One. 2021 Feb 9;16(2):e0244868. doi: 10.1371/journal.pone.0244868 (PMC7872272; doi:10.1371/journal.pone.0244868)
Supplement: S1 Table — (DOCX) [file pone.0244868.s001.docx]

**SURVEY**

**1. How many years have you been performing surgery?**

<5

5-10

11-20

21-30

>30

#### 2. What is your surgical subspecialty?


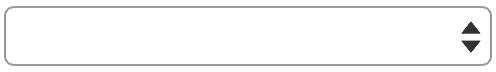


#### 3. What position do you most commonly take when operating?

Open surgery: standing

Microscopic surgery: sitting

Microscopic surgery: standing

Endoscopic surgery: sitting

Endoscopic surgery: standing

Robotic surgery: sitting at console

#### 4. Have you had significant discomfort while operating? If **NO**, **please respond so and jump to Question 12.**

Cervical neck pain

Lumbar pain

Both cervical and lumbar pain

Limb (shoulders, arms, legs, etc.)

No pain

#### 5. What activity causes you back pain? (Check all that apply)

Sitting during surgery

Standing during surgery

Sitting in clinic

Standing in clinic

None of these activities cause back pain

Other (please specify)

#### 6. What activity causes you the most back pain?

Sitting during surgery

Standing during surgery

Sitting in clinic

Standing in clinic

None of these activities cause back pain

Other (please specify)

#### 7. Average severity of your back pain during surgery or in clinic (10 is most severe)


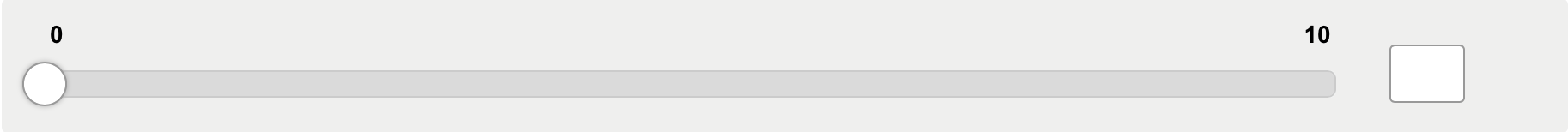


#### 8. Peak severity of your back pain during surgery or in clinic (10 is most severe)


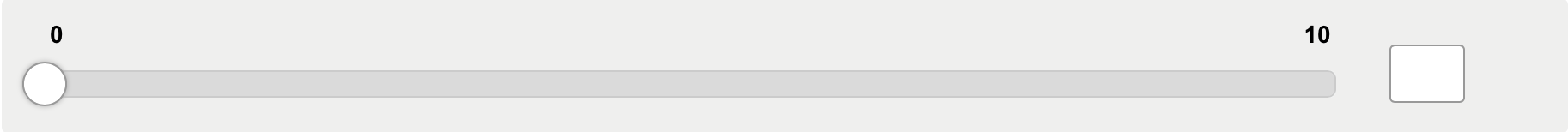


#### 9. What is the frequency with which you experience this pain during surgery or in clinic?

Less than once per week

1-2 times per week (occasionally)

3 or more times per week (commonly)

Continuously

#### 10. How severely has your discomfort/pain affected your ability to work as a surgeon?

Mildly

Moderately

Severely

Not at all

#### 11. How severely has your discomfort/pain affected physical activity outside of work?

Mildly

Moderately

Severely

Not at all

#### 12. In clinic and in the operating room, do you have any of the following ergonomic pieces of furniture? (check all that apply)

Outpatient clinic chair/stool

Surgical chair/stool

Office desk chair

Desk with up and down control

Other (please specify)

#### 13. Have you received any ergonomics training in the past?

Never

During medical school

During residency training

CME training

Expert consultation

Other (please specify)

#### 14. What is your gender?

Male

Female
