# Supplementary material for: The risk of ergonomic injury across surgical specialties
Source: PLoS One. 2021 Feb 9;16(2):e0244868. doi: 10.1371/journal.pone.0244868 (PMC7872272; doi:10.1371/journal.pone.0244868)
Supplement: Attachment — Submitted filename: comments.docx [file pone.0244868.s002.docx]

This manuscript adds some unique understand of the risk of ergonomic injury across surgical specialties. I only have some minor comments.

In your method, it is not clear if the experience the key factor of ergonomic included in the study. And it’s also not clear if the participants were always willing to be observed.
