# Supplementary material for: The risk of ergonomic injury across surgical specialties
Source: PLoS One. 2021 Feb 9;16(2):e0244868. doi: 10.1371/journal.pone.0244868 (PMC7872272; doi:10.1371/journal.pone.0244868)
Supplement: Attachment — Submitted filename: Response to Reviewers.docx [file pone.0244868.s004.docx]

*Dear PLOS Editorial and Reviewer Committee,*

*Thank you very much for taking the time to read our manuscript, and for your thoughtful and insightful feedback. Please see below for an itemized, point-by-point response to each of your comments. All changes in the manuscript are highlighted. We hope you find our revised version meeting the high standards of PLOS in consideration for publication*.

**Reviewer #1:**

1. I think that this manuscript adds some unique understand of the risk of ergonomic injury across surgical specialties. I thank the authors for their accurate and complete answers.

*Dear reviewer, our authors would like to sincerely thank you for reviewing our revision manuscript again and thank you for your feedback.*

**Review #3**

*Dear reviewer, thank you for thoroughly going over our revision manuscript and your astute suggestions. Please find a point by point response to your concerns that need addressing. We hope that this new version will meet the standard for publication in PLOS.*

1. Linguistically, some improvements still need to be made. Some examples: lines 70-72 (the word "find" used several times); lines 200-229 (the word "respectivly" is used very often - finding synonyms); line 310 spelling mistakes

*Thank you for pointing this out. The following changes were made within the manuscript:*

**Lines 70-72:** One such study objectively assessed intraoperative ergonomics using Rapid Upper Limb Assessment (RULA) and observed that 0% (0/275) of pediatric otolaryngologists were found to have a negligible level of ergonomic risk.

**Line 80-82** …. through observing Otolaryngologists at our institution and examining how objective and subjective scores correlated to ergonomic hazards.

*We have also changed the following sentences to assure that no word is repeated multiple times within consecutive sentences:*

**Lines 221**: Otolaryngologists (n=15, 31.3%) and ophthalmologists (n=8, 20.7%) noted experiencing discomfort in the cervical neck region while operating.

**Line 235-237**: The average difference in REBA scores for standing surgery were significantly higher than those for sitting (6.65 vs. 3.35, p<0.0001).

**Line 301 (we could not find a grammatical error)**

2. Line 89: It is said that the study is unique, but this cannot be said with certainty. It may be that a comparable study has been conducted*.*

*Thank you for bringing this to our attention. We have changed this sentence.*

**Line 87-89:** Our manuscript is novel as it assesses both objective and subjective intraoperative ergonomic hazards and severity of MSK symptoms across multiple surgical specialties.

3. Line 109: Unclear why table 1 comes pages later?

*Thank you for your comment. We had originally placed the survey mentioned in line 109 at the end of the manuscript because it was a supplemental table. We have now put this immediately after line 116 for clarity.*

4. Line 122: here, the authors are clearly referred to. Anonymity is therefore not guaranteed.

*Thank you for pointing this out, it was not our intention to de-anonymize the manuscript. We will be more careful moving forward to assure this does not happen.*

*5.* Line 128: the reference to figure 1 is made, but you don't see the image in the text and I couldn't find it this way

*Thank you for this comment and pointing this out. The figures were separately included as requested by the journal and are available at the end of the manuscript in the pdf version. We tried to adhere to the journal preferred arrangement of the Figures (submitted as a separate file) and Tables (embedded within the text following the paragraph where it is first mentioned).*

*6.* In general: locked spaces should be used to display the results. In addition, it is not always clear what the sample is that answered the corresponding questions (e.g. lines 165-170, lines 177-190)

*Thank you for your suggestion. Given that the journal has their own standards for arranging the figures within the copy of the manuscript, we did not want to add lock spacing to our results, thus allowing the editors and the production team to be able to arrange them as they best see fit.*

*In order to clarify the sample size, we have changed the following:*

**Lines 165-172:** There were 155 participants that responded to questions on ergonomics knowledge (Table 1). Of those 155, 118 (76.1%) surgeons had no prior ergonomics training while 37 had some ergonomics training prior to the study. Eight (5.2%) stated that training occurred during medical school, 13 (8.4%) received it while in residency, 3 (1.9%) obtained it from an expert consultation, while another 13 (8.4%) reported other means of receiving training.

In regard to ergonomic accessibility, out of 167 surgeons, 141 (84.4%) stated that they did not have access to ergonomic chair/stools in their clinic. Furthermore,120 (71.8%) surgeons reported no access to this equipment in the operating room.

7. Line 156: unclear why eight out of ten programs were selected?

*Thank you for your question. We sent the survey to ten different programs, but we received no response from any surgeon from two distinct programs. We have changed the sentence to further clarify this.*

**Line 157-158:** Out of the 389 surgeons that received the survey, 167 (42.9%) responded across eight out of ten programs that the surveys were sent to.

8. Table 1: very confusing, because a lot has been summarized here.

*Thank you for your concern, this table was implemented during the first revision version as requested from comments by other reviewers. The data within the table corresponds to the questions that were asked in the survey (supplemental table 1). We feel that it is easy to follow, as it directly summarizes the results from the survey. Unless you strongly feel that the table should not be included, we would like to keep the table as is. Or welcome suggestions to how it might more clearly present the summarized data.*

9. Row 275: here the only time reference is made to concrete authors by name. Inconsistent with the rest of the style

*Thank you for your insightful observation. We have changed this sentence and it now reads:*

**Line 274-277:** This is in line with a recently published meta-analysis that examined a total of 5152 surveyed surgeons looking at reported pain across multiple surgical subspecialties and finding that back (50%), neck (48%), and arm or shoulder (43%) pain was the most common. [3]

10. Limitations from line 345: unclear which limitations are addressed? If the reader is not familiar with the language, he does not know what the language is about. Literature and concrete description are missing

*Thank you for your comment. We have revised this line to specifically address the limitation and have cited the appropriate literature.*

**Line 344-345**: We acknowledge that certain aspects of our study, such as subjective MSK symptoms and outcomes, have been studied in other medical specialties. [3]

*Additionally, we have added citations and briefly elaborated on the suggested alternative methodologies that can be implemented as seen below:*

**Line 346-352** In order to evaluate the actual MSK load of the surgeon during the operation, other tools such as surface electromyography (to be able to estimate dynamic force or fatigue during a tasks), [24] simulation-based ergonomics training curriculum, [25] and other instrumented and assessment tools, would be more meaningful - especially as it relates to evaluating the underlying MSK disorders in this surgical cohort. An additional limitation is not having an interpersonal correlation between reported symptoms on the survey and intraoperative REBA observations, due to the anonymous nature of the survey.

**Reviewer #4:**

*Dear reviewer, thank you for reviewing our revision manuscript again and thank you for your original feedback.*
